# Non-timely clinically applicable ADC ratio in prostate mpMRI: a comparison with fusion biopsy results

**DOI:** 10.1007/s00261-022-03627-w

**Published:** 2022-08-09

**Authors:** Zeno Falaschi, Stefano Tricca, Silvia Attanasio, Michele Billia, Chiara Airoldi, Ilaria Percivale, Simone Bor, Davide Perri, Alessandro Volpe, Alessandro Carriero

**Affiliations:** 1 Department of Diagnosis and Treatment Services, Radiodiagnostics, Azienda Ospedaliero Universitaria Maggiore della Carità, Corso Mazzini 18, 28100 Novara, Italy; 2 Department of Urology, Azienda Ospedaliero Universitaria Maggiore della Carità, Corso Mazzini 18, 28100 Novara, Italy

**Keywords:** Prostate cancer, Multiparametric MRI, Apparent diffusion coefficient, Gleason score

## Abstract

**Purpose:**

The purpose of the study was to assess the diagnostic accuracy of ADC ratio and to evaluate its efficacy in reducing the number of false positives in prostatic mpMRI.

**Materials and methods:**

All patients who underwent an mpMRI and a targeted fusion biopsy in our institution from 2016 to 2021 were retrospectively selected. Two experienced readers (R1 and R2) independently evaluated the images, blindly to biopsy results. The radiologists assessed the ADC ratios by tracing a circular 10 mm^2^ ROI on the biopsied lesion and on the apparently benign contralateral parenchyma. Prostate cancers were divided into non-clinically significant (nsPC, Gleason score = 6) and clinically significant (sPC, Gleason score ≥ 7). ROC analyses were performed.

**Results:**

167 patients and188 lesions were included. Concordance was 0.62 according to Cohen’s K. ADC ratio showed an AUC for PCAs of 0.78 in R1 and 0.8 in R2. The AUC for sPC was 0.85 in R1 and 0.84 in R2. The 100% sensitivity cut-off for sPCs was 0.65 (specificity 25.6%) in R1 and 0.66 (specificity 27.4%) in R2. Forty-three benign or not clinically significant lesions were above the 0.65 threshold in R1; 46 were above the 0.66 cut-off in R2. This would have allowed to avoid an equal number of unnecessary biopsies at the cost of 2 nsPCs in R1 and one nsPC in R2.

**Conclusion:**

In our sample, the ADC ratio was a useful and accurate tool that could potentially reduce the number of false positives in mpMRI.

**Graphical abstract:**

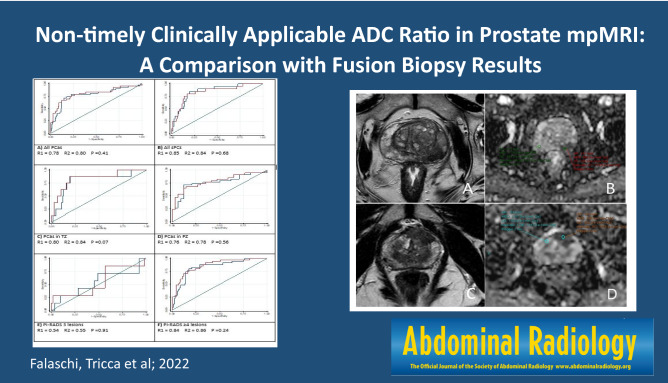

## Introduction

Prostate cancer (PCa) is one of the most common neoplastic diseases in men. The disease is expected to affect approximately 248,000 men and cause 34,000 deaths in the USA in 2021 [1]. The definitive diagnosis of PCa is dependent on the recognition of cancer cells in a tissue biopsy [2]. Multiparametric prostate magnetic resonance imaging (mpMRI) is the standard imaging technique to visualize PCa [3]. Prostate Imaging Reporting and Data System version 2 (PI-RADS 2) and 2.1 (PI-RADS 2.1) are the most used reporting methods [4, 5].PI-RADS v2 and 2.1 demonstrated an optimal diagnostic accuracy in PCa detection. A recent meta-analysis reported an overall PI-RADS 2 sensitivity of 0.85 and a specificity of 0.71 [6]. Moreover, mpMRI has proven to be better than prostatic specific antigen (PSA) alone in selecting the patients to biopsy [7]. Still the relatively low specificity and positive predictive value (PPV) lead to a significant number of false positives. These men often undergo an unnecessary biopsy with the related costs and complications of the procedure.

PI-RADS v2.1 suggests diffusion-weighted imaging (DWI) and quantitative apparent diffusion coefficient (ADC) maps are the most significant sequences of mpMRI for prostate cancer detection in the peripheral zone (PZ) [8]. DWI and ADC maps have also significant value in the transitional zone (TZ), although they are not the most important sequences to assess. The fact that lesions with lower ADC values correspond to prostate cancers with higher histologic Gleason Score (GS) is widely accepted in current literature [9]. ADC is not an absolute but an “apparent” value. It varies according to MRI scanner type, vendor, used MRI protocol, room temperature, patient, and coil. Therefore, normalizing ADC by dividing it by a reference tissue such as the bladder wall, urine, muscle, or the benign prostatic parenchyma has been proposed several times [10–12]. The concept is known as ADC ratio. ADC ratio may help to overcome the inherent ADC variability. On the other hand the rather subjective choice of where to position both the regions of interest (ROI) may generate further irregularity. In fact, the ADC ratio is currently not included in PI-RADS v 2.1, and the widely held opinion is that it needs additional verification [13, 14].

In 2020 a study [15] tried to assess whether ADC ratio may be helpful in reducing the number of mpMRI false positives, but they used the relatively imprecise 12-core standard prostatic biopsy (SB) as standard of reference. In a recent meta-analysis, fusion transrectal ultrasound-MRI targeted biopsy (fusion prostate biopsy, FPB) demonstrated a better sensitivity for significant prostate cancers (sPCa) diagnosis [16].The purpose of this study was to evaluate whether ADC ratio can be helpful in reducing the number of false positives in mpMRI in clinical practice, adopting FPB as standard of reference. We focused on the apparently benign contralateral parenchyma to calculate the ADC ratio and we used only the simplest methods that can be replicated by everyone in the clinical practice.

## Materials and methods

### Patient population and study design

This retrospective study complied with the guidelines issued by the Institute Research Medical Ethics Committee at our institution and was conducted in accordance with the declaration of Helsinki. Written informed consent was waived.

**Fig. 1:**
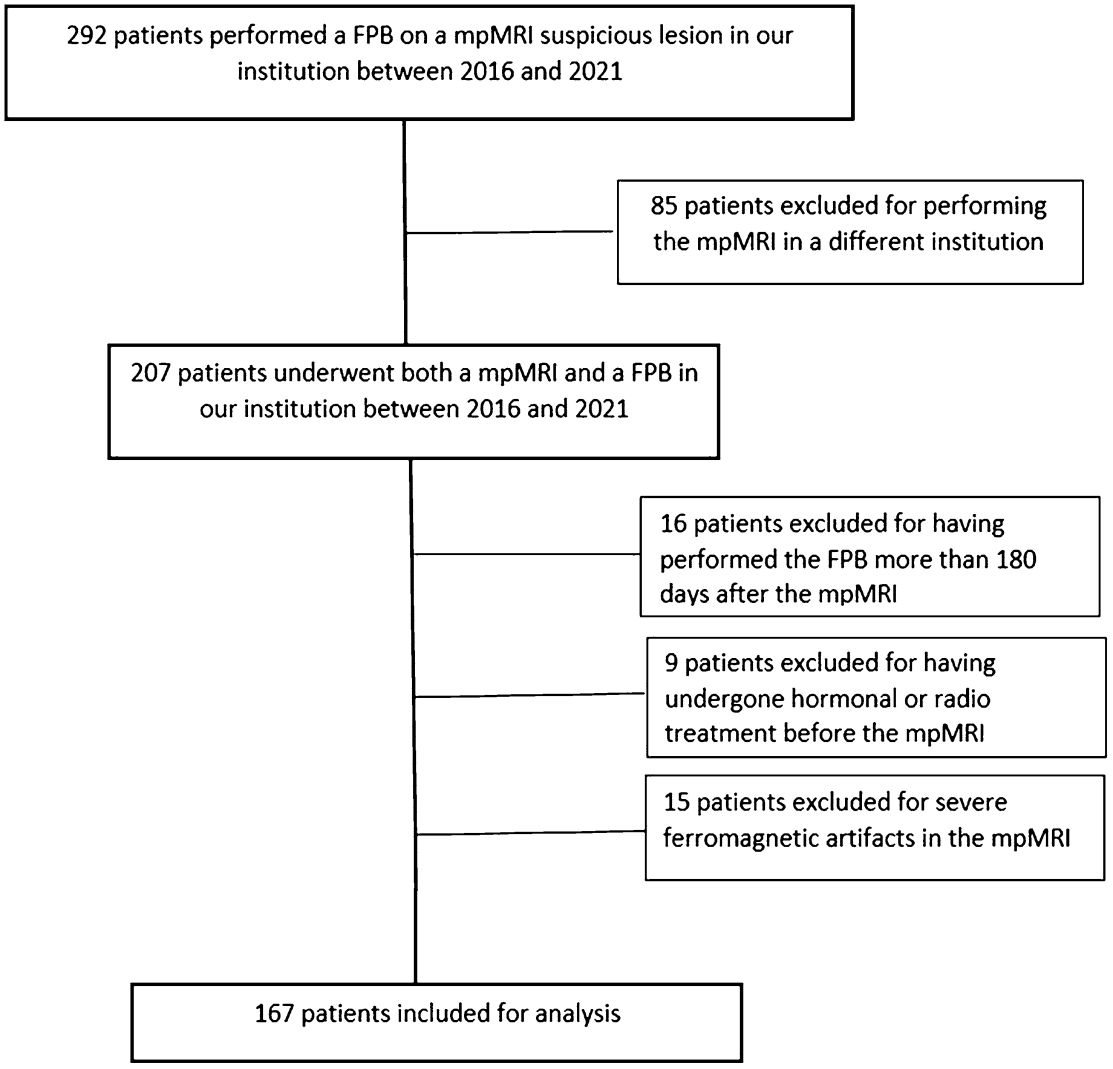
Flowchart of the study. FPB, fusion prostate biopsy; mpMRI, multiparametric prostate MRI

This single-center study included a series of 167 men who underwent both mpMRI and FBP in our institution between January 2016 and March 2021. Patients were enrolled retrospectively through the picture archiving and communication system and the archive of histopathological reports of our hospital. A flowchart of patients selection is shown in Fig. 1.

The patient inclusion criteria were as follows:

1. Having performed a 1.5T or 3.0T prostate mpMRI at our department;
2. Presence of at least one prostatic lesion with a PI-RADS assessment category of 3 or more made by the reporting radiologist;
3. Having undergone a FPB at our institution no more than 180 days after the mpMRI;

The exclusion criteria were as follows:

1. Previous invasive treatment or pharmacological therapies for PCa;
2. Incomplete mpMRI exam due to artifacts on MRI images or premature ending of the study.

For each patient, personal data such as date of birth, mpMRI exam date, and FPB date were recorded.

Enrolled patients were assigned an identification number (ID), which was later used to identify the study members allowing anonymity and protection of sensitive data.

All MRI identified lesions were divided into benign formations, non-clinically significant prostate cancers (nsPC, GS 6 3 + 3) and clinically significant prostate cancers (sPC, GS ≥ 7 3 + 4) according to FPB results.

### Multiparametric MRI

mpMRI examinations were performed on a 1.5T or 3.0T scanner (Ingenia 1.5T or 3.0T, Philips Healthcare, Netherlands). mpMRI protocol included T2-weighted images in 3 planes, DWI with subsequent ADC maps and dynamic contrast-enhanced imaging. An external pelvic coil was used in all cases. We administered an antiperistaltic agent (hyoscine N-butylbromide 20 mg/mL, 1 ml) intravenously to all patients who had no contraindications in order to reduce motion artifacts.

The DWI technical parameters in the 1.5T scanner were FOV 199 × 295 × 82; TR range 2500–5000; TE 70 ms; flip angle 90°; slice thickness 3 mm with no inter-slice gap; voxel size 2.5 × 2.8 × 3 mm (acquisition) and 1.84 × 1.84 × 3 mm (reconstruction); B factors 0, 800, and 1600 s/mm^2^.

The DWI technical parameters in the 3.0T scanner were FOV 160 × 100 × 72; TR range 2500–8000; TE 70 ms; flip angle 90°; slice thickness 3 mm with no inter-slice gap; voxel size 2.5 × 2.8 × 3 mm (acquisition) and 0.71 × 0.71 × 3 mm (reconstruction); B factors 0, 800, and 1600 s/mm^2^.

### PI-RADS assessment

The reporting radiologist classified each suspicious lesion according to PI-RADS v2 [4] up to April 2019. Lesions from April 2019 to March 2021 were classified according to PI-RADS v2.1 [5, 17]. We included only the biopsied lesions that received a PI-RADS classification ≥ 3 in the analysis. Since the FPB was performed on the basis of the original report we did not carry out a PI-RADS revision by an additional radiologist.

### ADC ratio calculation and images revision

Two radiologists with 11 and 4 years of genitourinary imaging experience were indicated as independent readers (R1 and R2) and reviewed all MRI examinations using the departmental CARESTREAM Vue PACS (Carestream health Italia, Milano, Italy).

**Fig. 2:**
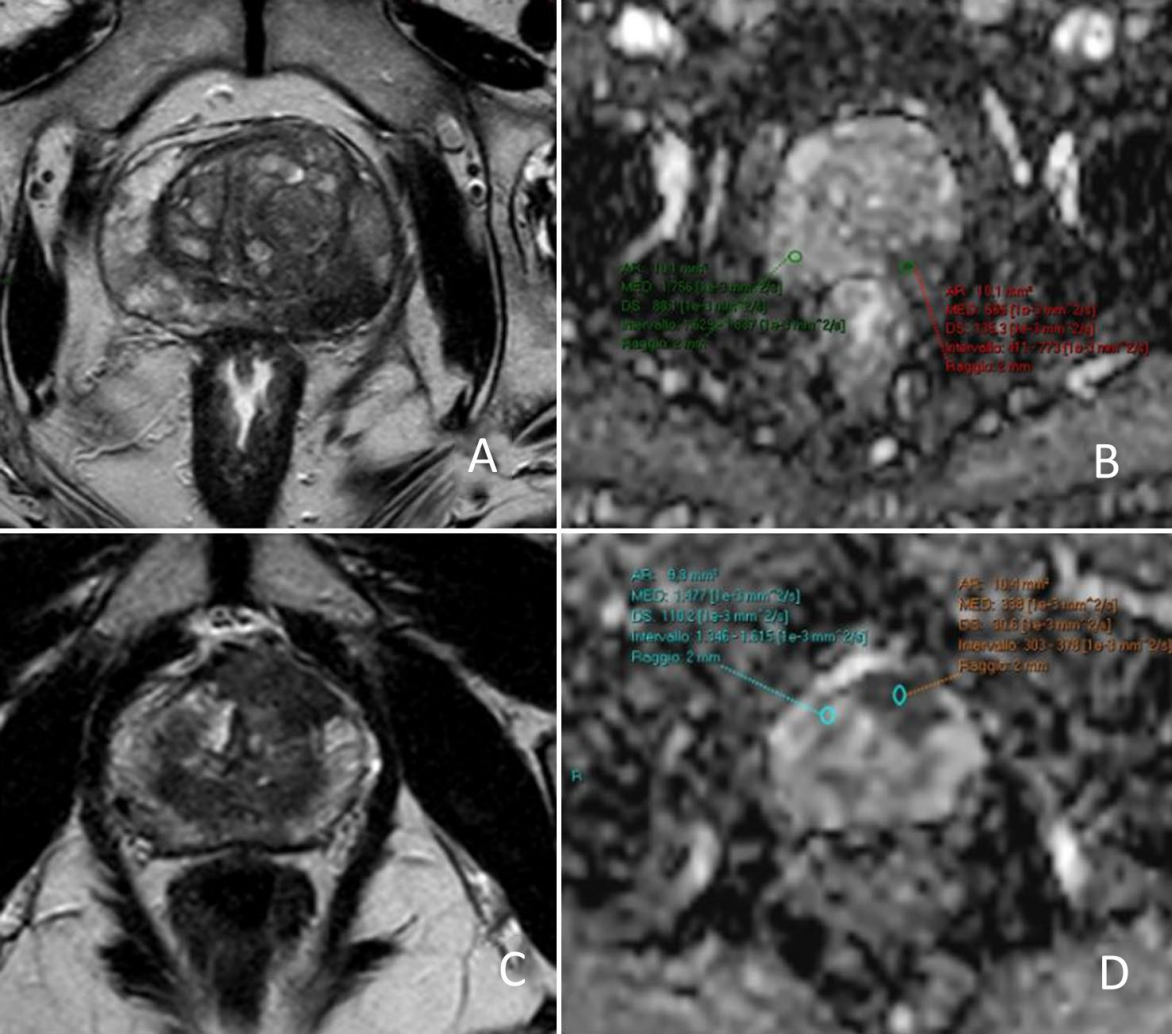
**a**, **b** ADC ratio calculation in a suspected PZ lesion in a 71 years old man. ADC ratio is 0.32 (565/1756). FPB later confirmed a PCa Gleason score 7 (4 + 3) in the area. **c**, **d** ADC ratio calculation in a suspected TZ lesion in a 70 year old man. ADC ratio was 0.22 (338/1477). FPB afterward confirmed a PCa Gleason score 8 (4 + 4) in the mpMRI signaled lesion. *ADC* apparent diffusion coefficient, *PZ* peripheral zone, *FPB* fusion prostate biopsy, *PCa* prostate cancer, *TZ* transitional zone, *mpMRI* multiparametric prostate MRI

Figure 2 shows an example of the ADC ratio calculation. Readers had the original report available in order to identify the target lesions, but were blinded to the histopathological results. They performed the ADC ratio calculation according to Bajgiran et al. and Falaschi et al. [12, 15]. Firstly, readers identified the biopsied lesion on the basis of both the radiology and the urology report. Afterward they drew a 10 mm^2^ circular region of interest (ROI) on the ADC map on the supposedly biopsied area. Since lowest ADC values are known to correspond to higher GS, the ROI was positioned on the darkest area within the lesion [18]. Moreover, readers placed a second 10 mm^2^ circular ROI in a portion of contralateral normal-appearing prostate parenchyma, in particular in the same prostate zone where the suspected lesion was located: peripheral zone (PZ) if the target lesion was in the PZ and transitional zone (TZ) if the lesion was in TZ. The mean ADC value in the lesion and the mean value in benign prostatic parenchyma were recorded. Finally, a software automatically divided the first by the second mean ADC values, thus obtaining the ADC ratio.

At the end of data collection, the cases in which the FPB was negative but a sPC was found in the standard biopsy were reviewed by both readers in consensus, assessing the most likely cause.

### Targeted fusion biopsy technique

Software-based MRI-targeted/TRUS fusion biopsies of the prostate followed by 12-core systematic biopsies were taken under loco-regional anesthesia in all patients by an expert urologist. Core biopsy samples were taken via 18G trucut disposable needle using a transrectal or transperineal approach based on surgeon’s preferences. Prior to the procedure fusion biopsy was planned using T2-weighted MRI images integrated into the Biojet dedicated software. Planning images were therefore superimposed to real time TRUS images in order to facilitate real time core specimen acquisition. Three core biopsy samples of each targeted lesion were obtained in all cases using end-fire ultrasound probe (BK), whereas standard mapping biopsy samples were taken using standard segmentation of the gland to acquire medial and lateral cores from each sextant prostate region. An outpatient procedure was performed in all cases.

### Statistical analysis

The minimum sample size was evaluated in relation to our primary aim: the ADC-ratio AUC estimate. We hypothesized: AUC = 0.80; L (the desired width of one-half of CI) = 0.1; k (non-malignant/malignant ratio) = 3.5; alpha error = 0.05. The total sample size, using an estimator of the variance function based on an underlying binormal distribution, was 131 (29 malignant and 102 non-malignant) [19].

We described continuous variables using means and standard deviation or median and interquartile range, as appropriate, while categorical ones were described using absolute and relative frequencies. First, we evaluated the inter-reader agreement among the two readers using the Bland Altman plot [20]. Then, in order to evaluate the concordance among the two radiologists, we divided the ADC ratios into three percentile categories (0–33, 34–66, 67+) and we calculated the weighted Cohen’s K with its 95% confidence interval (95% CI).

The difference between ADC values in exams performed on 1.5T and 3.0T scanner was evaluated in R1 measurements using Student T test.

Moreover, we used the FPB histologic results as gold reference for each biopsied lesion. The diagnostic accuracy of ADC ratio was evaluated in both readers using the area under the receiver operating characteristics curve (AUC).

All the subsequent analyses but the maximum sensitivity threshold for sPCs were performed on both readers. The AUC of the readers was calculated in:

- All PCas
- significant PCas
- PCas in the transitional zone (TZ)
- PCas in the peripheral zone (PZ)
- PI-RADS 3 lesions and PI-RADS ≥ 4 lesions

We tabulated the ADC ratio sensitivity, specificity, positive predictive value, and negative predictive value for all PCas and for sPC according to different possible cut-offs. We chose the optimal cut point according to maximum sensitivity for sPC.

Statistical analyses were performed with Stata 15 (StataCorp 2017).

## Results

### Population characteristics

Two-hundred-seven patients underwent an MpMRI and an FPB in our institution between January 2016 and March 2021. Sixteen patients were excluded for having undergone the FPB more than 180 days after the npMRI. Fifteen patients were excluded because of severe ferromagnetic artifacts on the MRI, most often because of hip prostheses. Finally, 9 patients who received hormonal therapy or radiotherapy prior to the mpMRI were excluded. One-hundred-sixty-seven (167) patients were included in the analysis. Figure 1 depicts patient selection.

Table 1 summarizes the population characteristics. The average patient age was 67 years, range 46–81. Median PSA in the cohort was 9.1 ng/mL (range 1.21–68.0). Digital rectal exploration was positive in 32 patients (19.1%). Ninety patients (53.9%) were biopsy naive while 77 (47.1) were re-biopsy candidates.

**Table 1:** Clinical data of the study population

Characteristic	Cohort (*n* = 167)
Median age (years)	66.9 (± 6.88)
Median PSA (ng/mL)	9.1 (± 6.9)
Positive Digital Rectal Examination (DRE) [*n* (%)]	32 (19.1%)
Per patient PCa found in all biopsies (SB + FBP) [*n* (%)]	59 (35.3%)
Targeted Biopsied Lesions	188
PCa found in FPB [*n* (%)]	42 (22.3%)
Peripheral Zone (PZ)	34 (80.9%)
Transitional Zone (TZ)	8 (19.0%)
sPCa found in FPB [*n* (%)]	20 (10.6%)
Peripheral Zone (PZ)	18 (90%)
Transitional Zone (TZ)	2 (10%)
Maximum GS in FPB [*n* (%)]	
No PCa	146 (77.6%)
Not evaluable	4 (2.1%)
6 (3 + 3)	18 (9.6%)
7a (3 + 4)	8 (4.3%)
7b (4 + 3)	9 (4.8%)
8 (4 + 4)	3 (1.6%)
9 (4 + 5)	0 (0%)
10 (5 + 5)	0 (0%)
sPCa found only in SB	11
mpMRI unseen lesions	5
FPB Gleason underscoring	4
FPB false negatives	2
Number of targeted biopsied lesions per patient [*n*(%)]	
1 lesion	146 (87.4%)
2 lesions	21 (12.6%)
Per patient MRI index lesion [*n* (%)]	
PIRADS 3	52 (31.1%)
PIRADS 4	75 (44.9%)
PIRADS 5	40 (24.0%)
MRI assessment per lesion [*n* (%)]	
PIRADS 3	60 (31.9%)
PIRADS 4	88 (46.8%)
PIRADS 5	40 (21.3%)
Lesions zone on MRI	
PZ	70 (37.2%)
TZ	118 (67.8%)
FPB cores median	2
SB cores median	12

*PSA* prostate specific antigen, *PCa* prostate cancer, *FPB* fusion prostate biopsy, *GS* Gleason score, *sPCA* significant PCA, *mpMRI* multiparametric prostate MRI

### Biopsy results and mpMRI revision

The 167 patients harbored 188 targetedly biopsied lesions. The urologist performed a single targeted biopsy in 146 patients (86.4%), while 21 patients (12.6%) received two targeted biopsies. No patients underwent more than two targeted biopsies.

The fusion biopsy was positive for PCa in 42 lesions (22.3%) found in 38 different patients (22.8%). A sPC was diagnosed in 20 targetedly biopsied lesions (10.6%).

The distribution of the positive FPB lesions is shown in Table 1. Noteworthy, only 2 sPCs were found in the TZ, while the radiologists assigned a PI-RADS 3 or higher classification to 118 TZ lesions. In comparison the FPB found 18 sPCs in the PZ in view of 68 mpMRI signaled lesions. The same table displays the Gleason score in positive FPBs.

The PI-RADS classification originally assigned to PCas and sPCs is shown in Table 2.

**Table 2:** PI-RADS classification originally assigned to PCas and sPCs

(A)		
PI-RADS	True positives	False positives
3	7	53
4	18	70
5	17	23
Total	42	146

(B)			
PI-RADS	PPV	Sensitivity	Specificity
≥ 3	22.3% (95% CI 16.6–29.0)	Not applicable	Not applicable
≥ 4	27.6% (95% CI 19.8–35.3)	83.3% (95% CI 72.1–94.6)	36.6% (95% CI 28.7–44.4)
5	42.5% (95% CI 27.2–57.8)	40.5% (95% CI 25.6–55.3)	84.1% (95% CI 78.2–90.1)

### ADC ratio

There was no statistically significant difference between ADC values obtained from 1.5T and 3.0T scanner. The mean ADC value for reported lesions was 837.7 ± 72.9 in 1.5T and 845.1 ± 61.7 in 3.0T, *p* = 0.29. The mean ADC value for benign parenchyma in 1.5T and 3.0T were 1601.7 ± 98.3 and 1589.4 ± 112.9, respectively, *p* = 0.26.

**Fig. 3:**
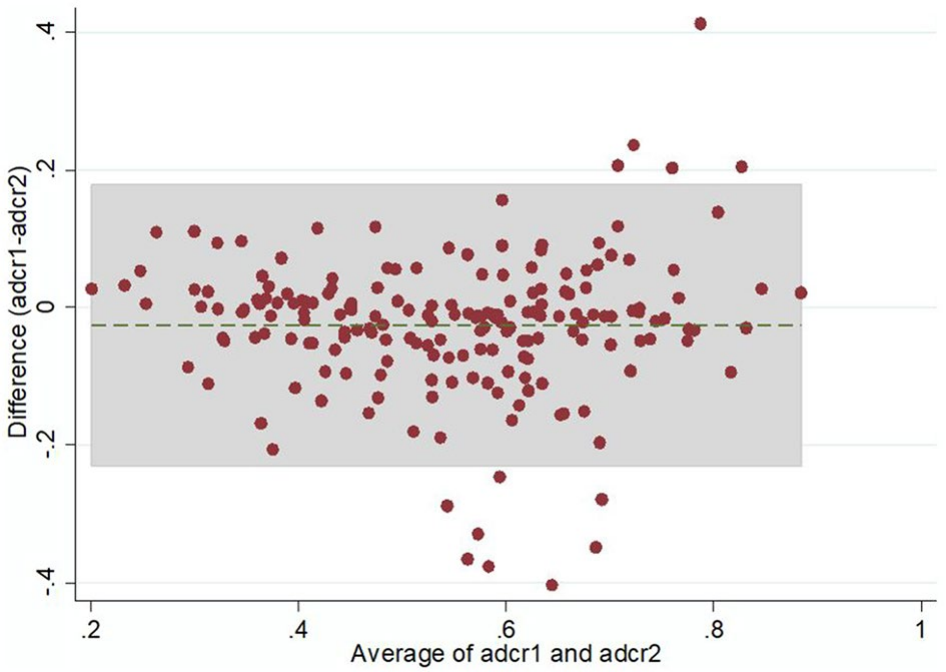
Bland Altman plot showing agreement between the two readers. adcr1, apparent diffusion coefficient ratio—reader 1; adcr2, apparent diffusion coefficient ratio—reader 2

Inter-reader agreement evaluated with Bland Altman plot [20] is shown in Fig. 3. The analysis performed with Cohen’s K resulted in an agreement of 82.7% between the two readers. Cohen’s K was 0.62 (CI 0.52–0.69), corresponding to a moderate to substantial agreement according to Landis and Koch [21].

**Fig. 4:**
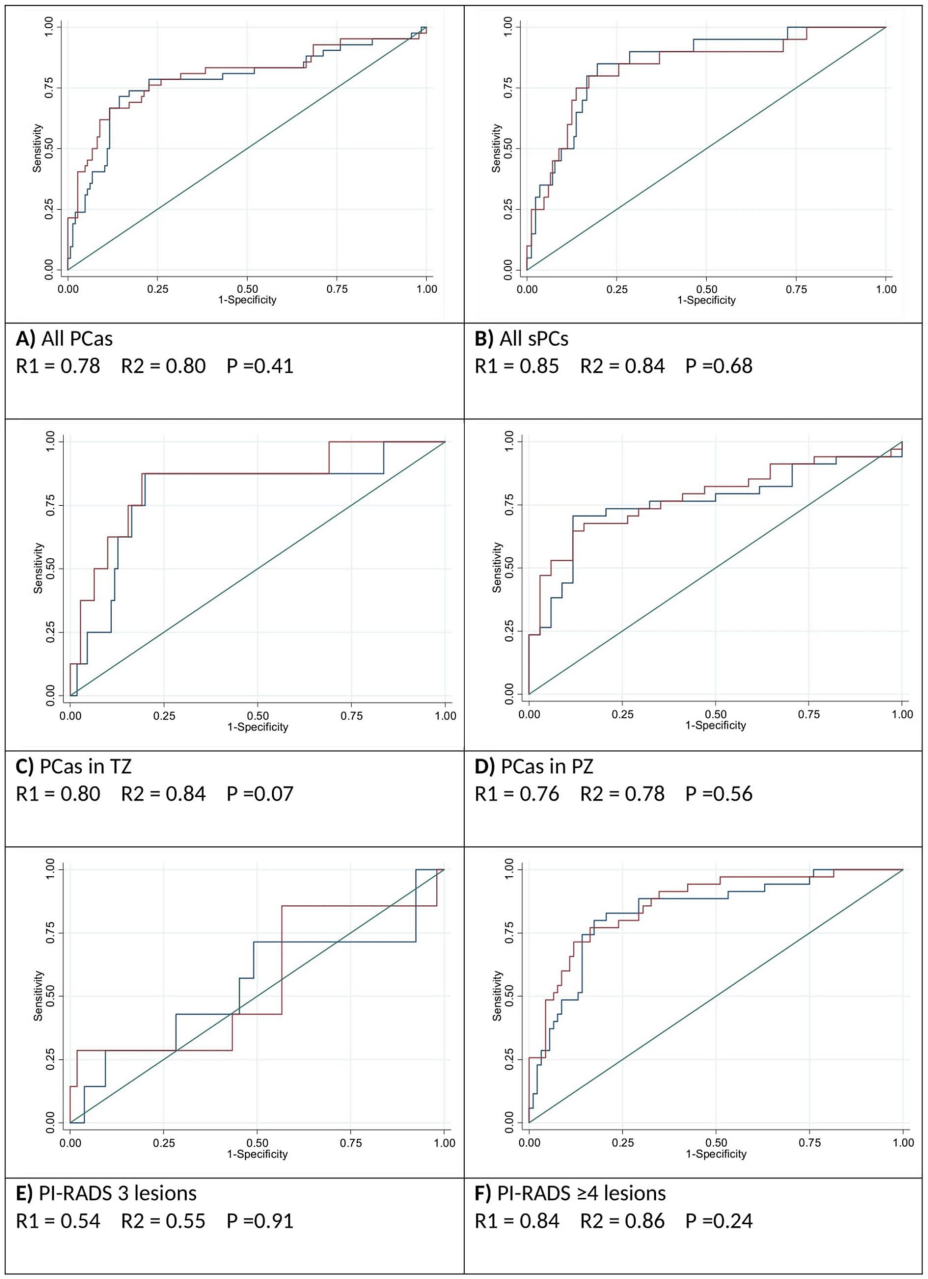
Blue lines represent R1 and red lines represent R2. **a** ADC ratio ROC curves for all PCas. The AUCs are 0.78 in reader 1 and 0.8 in reader 2. **b** ADC ratio ROC curves for sPCs. **c** ADC ratio ROC curves for all PCas in TZ. **d** ADC ratio ROC curves for all PCas in PZ. **e** ADC ratio ROC curves for all PCas in PI-RADS 3 category. **f** ADC ratio ROC curves for all PCas in PI-RADS ≥ 4 categories. *ADC* apparent diffusion coefficient, *ROC* receiver operating characteristics, *AUC* area under the curve, *PCa* prostate cancer, *sPC* clinically significant prostate cancer, *TZ* transitional zone, *PZ* peripheral zone

Figure 4 displays ADC ratio ROC curves in reader 1 and reader 2 according to PCa diagnosis made by FBP. The AUCs were 0.78 (95% CI 0.69–0.87) in reader 1 and 0.8 (95% CI 0.71–0.89) in reader 2. Table 3 shows different possible ADC ratio cut-offs for the PCa diagnosis in reader 1.

**Table 3:** Diagnostic accuracy of ADC ratio for all PCas at different cut points in reader 1

Cut-point	SENS	CI	SPEC	CI	PPV	CI	NPV	CI
0.30	0.14	0.07 0.23	0.99	0.95 1.00	0.75	0.41 0.93	0.80	0.74 0.85
0.35	0.31	0.19 0.46	0.95	0.90 0.98	0.65	0.43 0.82	0.83	0.76 0.88
0.40	0.62	0.47 0.75	0.88	0.82 0.93	0.60	0.46 0.74	0.89	0.83 0.93
0.45	0.74	0.59 0.85	0.78	0.71 0.84	0.49	0.37 0.61	0.91	0.85 0.95
0.50	0.79	0.64 0.88	0.67	0.59 0.74	0.41	0.31 0.52	0.92	0.85 0.95
0.55	0.81	0.67 0.90	0.53	0.45 0.61	0.33	0.25 0.43	0.91	0.83 0.95
0.60	0.83	0.69 0.92	0.36	0.28 0.44	0.27	0.20 0.35	0.88	0.78 0.94
0.65	0.90	0.78 0.96	0.27	0.20 0.34	0.26	0.20 0.34	0.91	0.78 0.96
0.70	0.93	0.81 0.98	0.17	0.12 0.24	0.24	0.18 0.32	0.89	0.73 0.96
0.75	0.95	0.84 0.99	0.10	0.06 0.16	0.23	0.18 0.30	0.88	0.66 0.97

*SENS* sensitivity, *SPEC* specificity, *PPV* positive predicted value, *NPV* negative predicted value, *95% CI *confidence interval according to the Wilson Method, *ADC* apparent diffusion coefficient, *PCa* prostate cancer

The ROC curve analyses for TZ, PZ, PI-RADS 3 lesions, and PI-RADS ≥ 4 lesions in both readers are shown in Fig. 4. The AUC is slightly higher in the TZ than in the PZ, albeit the number of cancers in TZ is inferior. The ROC curve analysis for PI-RADS 3 lesions reveals that ADC ratio is inaccurate in this category, with an AUC of 0.54 and 0.55 in R1 and R2, respectively. Indeed, seven FPB-confirmed PCas and 53 benign lesions were included in the PI-RADS 3 category. The PCas were found to be 5 nsPCs and 2 sPCs.

The B image in Fig. 4 indicates the ROC curve of sPCs only. The AUCs in this last category are 0.85 and 0.84 in R1 and R2. Table 4 displays different possible cut-offs for sPC diagnosis by ADC ratio. The maximum sensitivity threshold for sPC was 0.65 (sensitivity 100%, specificity 25.6%, PPV 13.8%, NPV 100%) in R1 and 0.66 (sensitivity 100%, specificity 27.4%, PPV 14.1%, NPV 100%) in R2. If all the lesions with an ADC ratio > 0.65 were not signaled, 43 unnecessary biopsies (43/168, 25.6%) could have been avoided according to Reader 1. According to Reader 2 if lesions with an ADC ratio > 0.66 were not signaled 46 unnecessary biopsies (43/168, 27.4%) could have been averted. The price would have been the misdiagnosis of two Gleason 6 PCas in Reader 1 and one Gleason 6 PCa in Reader 2.

**Table 4:** Diagnostic accuracy of ADC ratio for sPCs at different cut points in both readers

Reader	Cutpoint	SENS	CI	SPEC	CI	PPV	CI	NPV	CI
1	0.30	0.20	0.08 0.42	0.98	0.94 0.99	0.50	0.21 0.78	0.91	0.86 0.94
1	0.35	0.40	0.22 0.61	0.93	0.88 0.96	0.40	0.22 0.61	0.93	0.88 0.96
1	0.40	0.75	0.53 0.89	0.83	0.77 0.88	0.35	0.22 0.50	0.97	0.92 0.98
1	0.45	0.85	0.64 0.95	0.73	0.65 0.79	0.27	0.18 0.39	0.986	0.93 0.99
1	0.50	0.90	0.70 0.98	0.64	0.55 0.70	0.22	0.14 0.32	0.98	0.93 0.100
1	0.55	0.95	0.76 0.99	0.51	0.43 0.58	0.19	0.12 0.27	0.98	0.94 0.100
1	0.60	0.95	0.76 0.99	0.35	0.28 0.42	0.15	0.10 0.22	0.98	0.91 0.100
**1**	**0.65**	**1**	**0.84 1.00**	**0.26**	**0.20 0.33**	**0.14**	**0.09 0.20**	**1**	**0.92 1.000**
1	0.70	1	0.84 1.00	0.17	0.12 0.23	0.12	0.08 0.18	1	0.88 1.000
1	0.75	1	0.84 1.00	0.10	0.06 0.16	0.12	0.08 0.17	1	0.82 1.000
2	0.30	0.25	0.09 0.49	0.99	0.96 1.000	0.71	0.34 0.92	0.91	0.90 0.93
2	0.35	0.35	0.15 0.59	0.95	0.91 0.98	0.47	0.26 0.68	0.92	0.90 0.94
2	0.40	0.60	0.36 0.81	0.89	0.84 0.94	0.40	0.27 0.54	0.95	0.92 0.97
2	0.45	0.80	0.56 0.94	0.83	0.76 0.88	0.36	0.37 0.45	0.97	0.94 0.99
2	0.50	0.85	0.62 0.97	0.74	0.66 0.80	0.28	0.22 0.35	0.98	0.94 0.99
2	0.55	0.90	0.68 0.99	0.65	0.58 0.73	0.24	0.19 0.29	0.98	0.94 0.99
2	0.60	0.90	0.68 0.99	0.50	0.42 0.58	0.17	0.15 0.21	0.98	0.92 0.99
**2**	**0.66**	**1**	**0.83 1.00**	**0.27**	**0.21 0.35**	**0.14**	**0.13 0.15**	**1**	**0.92 1.000**
2	0.70	1	0.83 1.00	0.20	0.14 0.26	0.13	0.12 0.14	1	–
2	0.75	1	0.83 1.00	0.11	0.07 0.17	0.12	0.11 0.12	1	–

Bold indicates the cut-off values with maximum sensitivity value for the two readers

*SENS* sensitivity, *SPEC* specificity, *PPV* positive predicted value, *NPV* negative predicted value, *95% CI *confidence interval according to the Wilson Method, *ADC* apparent diffusion coefficient, *sPC* clinically significant prostate cancer

## Discussion

The potential role of ADC ratio in reducing the number of false positives in mpMRI and avoiding biopsies was investigated. The test proved to be accurate, as demonstrated by the ROC curves. We were able to identify a maximum sensitivity cutpoint for clinically significant prostate cancers.

This study aimed to implement the results previously obtained by Falaschi et al. [15] using the more accurate FPB as standard of reference. That study demonstrated an elevated diagnostic accuracy of the ADC ratio obtained with two circular 10 mm^2^ ROIs, and was able to identify a threshold at which 100% sensitivity was granted. The only few cases where FPB resulted negative but a sPC was found in the standard biopsy may be due to FBP Gleason underscoring, FPB missing the lesion or mpMRI not having seen the tumor, as in MRI invisible prostate cancers (see Table 1).

From a methodological point of view, for ADC measurements we chose to draw a circular 10 mm^2^ ROI also based on the work of Bajgiran et al. [12], who in his work drew multiple 10–20 mm^2^ ROIs on the ADC map to determine the minimum lesions’ values. Not only Bajgiran, but also other Authors like Moraes et al. [22] and Jyoti et al. [9] chose to place the ROI in the lowest ADC area. As regards the shape of the ROI, many Authors, like Bajgiran himself, Barrett et al. [23], and Pierre et al. [24] used a freehand ROI encompassing most of the tumor. Instead, Alessandrino et al. [14] used a circular ROI including most of the lesion and Wang et al. [10] drew a three-dimensional ROI encircling the entirety of the MRI suspected area.

Although less precise, we believe that in everyday clinical practice a 10 mm^2^ ROI is the easiest and most reproducible measurement. Specifically, free-hand drawing in our hand is more time consuming and less reproducible, leading to higher inter-reader variability. Three-dimensional ROIs are not available in every workstation, while we would like every researcher to be able to replicate our results. In accordance with the current literature, we were acknowledged that the diagnostic accuracy of mpMRI and of PI-RADS v2 and v2.1 were suboptimal and that a lower detection rate for TZ tumors is a limit of PI-RADS v2 [25–27]. Such a limits were obviously more impacting in daily clinical practice such as in our experience, resulting in a lot of false positives cases and unnecessary biopsies.

Our objective was to develop an instrument which was simple, reproducible, and rapid to perform in daily practice, and that could help the radiologists reduce the number of false positives. As a matter of fact, the inter-reader agreement resulted in moderate to substantial in our study, an improvement in comparison with the fair agreement registered by Pierre et al. [24], who used a free-hand drawn ROI. We believe that the biggest source of variability in our model is mainly the somewhat arbitrary decision of where to put the second “benign” ROI.

Moreover the AUCs in our investigation were appreciably higher than those reported in the similarly designed study by Wang et al. [10] thus indicating that our ADC ratio calculating method can be more accurate than a 3D ROI in the evaluation of the tumor malignity.

We think that the better accuracy obtained in comparison with Falaschi et al. [15] is due to the fact that only the standard 12 core biopsy was used as reference of standard in that study. FPB is indeed unanimously considered superior in precisely identifying the mpMRI signaled lesions.

Our performance is somewhat in keeping with the results of Polanec [18], which considered only the ADC absolute value and used the in-bore MRI biopsy as standard of reference. Similar results were obtained by Boesen et al. and Jyoti et al. [9, 13], who both assessed ADC ratio capability of discriminating between Gleason 6 and Gleason ≥ 7 tumors. Boesen used a circular ROI in the center of the lesion and employed the whole mount prostate specimen as standard of reference. Jyoti placed a 5–10 mm^2^ ROI in each lesion where the ADC values were lower and used the fusion biopsy as standard of reference.

Notably we were able to identify a 100% sensitivity threshold in both readers. Although the ADC ratio diagnostic accuracy for sPCs is very high at a cut point around 0.4–0.45 in both readers (Table 4), we would preferably recommend a threshold at which not even a single significant cancer is missed. Therefore we calculated the ADC ratio cutoffs which allowed a 100% sensitivity in both readers. Those values could have prevented a significant number of unnecessary biopsies at the cost of an extremely limited number of nsPCs. Given the costs in terms of capital, discomfort and potential complications of every biopsy, we believe that these results are particularly relevant for clinical practice.

**Fig. 5:**
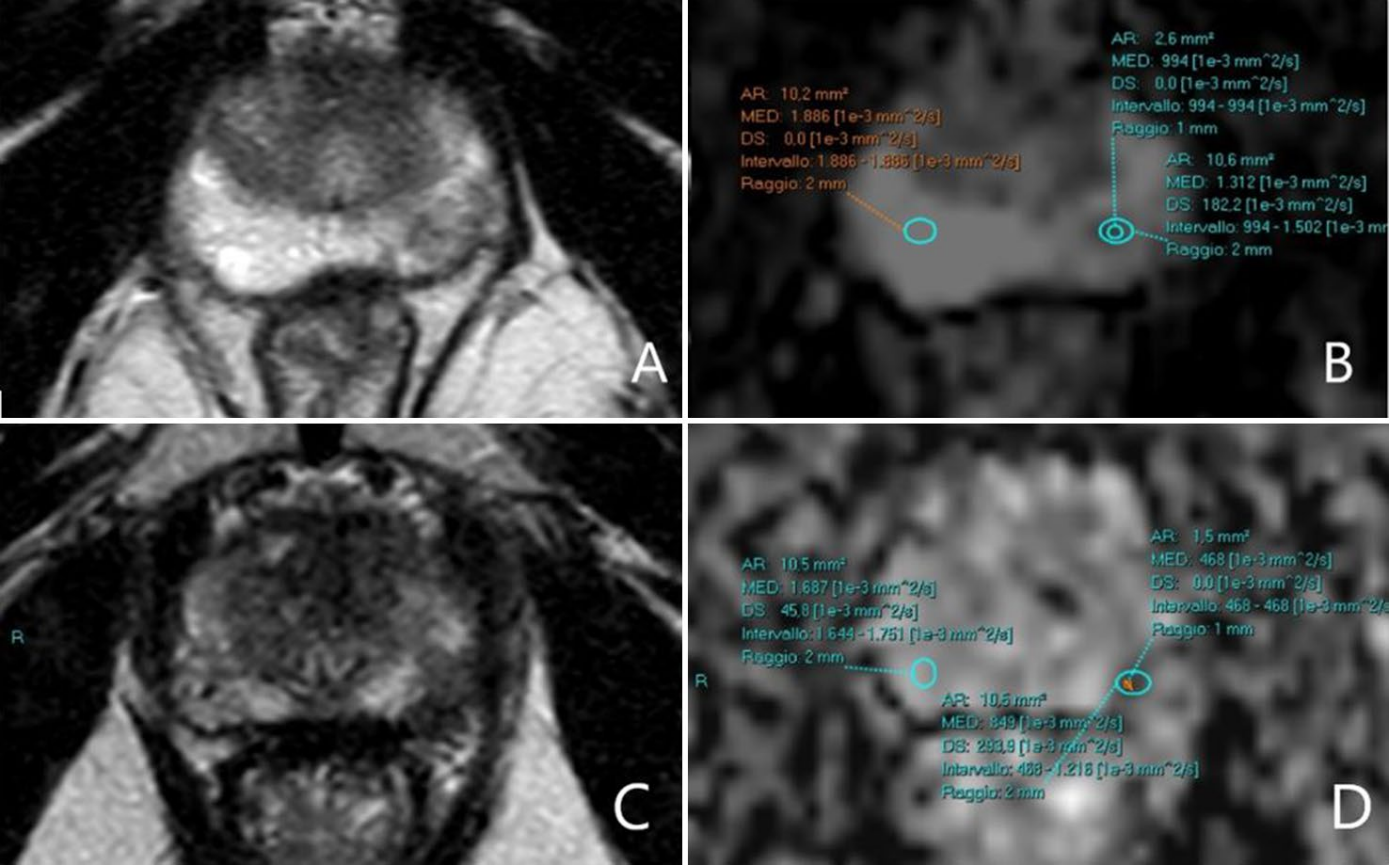
Two cases in which ADC ratio underperformed. **a**, **b** a 67 year old man with a FPB confirmed PCa Gleason 7 (3 + 4). The lesion was too small for a correct assessment with a 10 mm^2^ ROI. A smaller 2.6 mm^2^ ROI would have given a more correct assessment. **c**, **d** a 70 year old man with a SB confirmed PCa Gleason 7 (3 + 4), FPB was negative. In this case the lesion was so peripheral that both the measuring radiologist and the urologist missed it on the axial images. *ADC* apparent diffusion coefficient, *FPB* fusion prostate biopsy, *PCa* prostate cancer, *ROI* region of interest

On the other hand, we were unable to identify a 100% sensitivity threshold for all PCas. Some Gleason 6 lesions had elevated ADC values, and we believe this is the reason why the ADC ratio proved useless in identifying PCas in PI-RADS 3 lesions, while the accuracy in PI-RADS ≥ 4 was significant. Five PI-RADS 3 PCas were in fact GS 6 cancers, while the only clinically significant tumor (GS 7 3 + 4) was too small for correct assessment with a 10mm^2^ ROI, as shown in Fig. 5. In this picture are depicted two cases in which the ADC ratio proved unreliable. In particular we believe that our methodology should not be performed in lesions smaller than 10mm^2^, since the inclusion of benign parenchyma in the ROI can result in a false negative, and that our assessment is inaccurate in extremely peripheral lesions which are unapparent in the axial ADC map.

On the basis of our results we can affirm that ADC ratio can be more useful in the diagnosis of sPCas (GS > 7) which are associated with tumor related morbidity and mortality needing therapy rather than in the detection of nsPCas (GS 6 or less) which can be treated with clinical surveillance only [28, 29].

Our study has several limitations. The first one is that we used only the FPB as a standard of reference, not the whole mount histology or the combination of FPB and SB. We made this decision because we wanted to be as sure as possible that the biopsy samples actually came from the measured lesions. A second limitation is that the readers subjectively interpreted the mpMRI and the biopsy reports in order to find the biopsied lesions, and so they could have not measured the actual biopsied area. Thirdly the mpMRI examinations came from two different scanners during a long period of time, and this certainly brought an ample degree of variability into the cohort. We chose to measure the ADC ratio in order to normalize this heterogeneous data and in particular to eliminate the inter-scanner variability [10, 11].

## Conclusion

The results in our sample suggest that ADC ratio could potentially be an accurate and reproducible tool in the diagnosis of clinically significant prostate cancer. ADC ratio in our single-center experience was safely used to reduce the number of false positives in the mpMRI of the prostate. More studies are obviously needed in order to validate our findings in other centers and extend the indication of the method.

## Abbreviations

PCa: Prostate cancer
mpMRI: Multiparametric prostate magnetic resonance imaging
PI-RADS: Prostate Imaging Reporting and Data System
PSA: Prostatic specific antigen
DWI: Diffusion-weighted imaging
ADC: Apparent diffusion coefficient
PZ: Peripheral zone
TZ: Transitional zone
GS: Gleason Score
ROI: Region of interest
SB: 12-Core standard prostatic biopsy
FPB: Fusion prostate biopsy
sPC: Clinically significant prostate cancer
nsPC: Non clinically significant prostate cancer
